# Changes in Neuronal Oscillations Accompany the Loss of Hippocampal LTP that Occurs in an Animal Model of Psychosis

**DOI:** 10.3389/fnbeh.2017.00036

**Published:** 2017-03-08

**Authors:** Alexander N. Kalweit, Bezhad Amanpour-Gharaei, Jens Colitti-Klausnitzer, Denise Manahan-Vaughan

**Affiliations:** ^1^Department of Neurophysiology, Medical Faculty, Ruhr University BochumBochum, Germany; ^2^International Graduate School of Neuroscience, Ruhr University BochumBochum, Germany

**Keywords:** psychosis, schizophrenia, first-episode psychosis, LTP, neuronal oscillations, rodent

## Abstract

The first-episode of psychosis is followed by a transient time-window of ca. 60 days during which therapeutic interventions have a higher likelihood of being effective than interventions that are started with a greater latency. This suggests that, in the immediate time-period after first-episode psychosis, functional changes occur in the brain that render it increasingly resistant to intervention. The precise mechanistic nature of these changes is unclear, but at the cognitive level, sensory and hippocampus-based dysfunctions become increasingly manifest. In an animal model of first-episode psychosis that comprises acute treatment of rats with the irreversible N-methyl-D-aspartate receptor (NMDAR)-antagonist, MK801, acute but also chronic deficits in long-term potentiation (LTP) and spatial memory occur. Neuronal oscillations, especially in the form of information transfer through θ and γ frequency oscillations are an intrinsic component of normal information processing in the hippocampus. Changes in θ-γ coupling and power are known to accompany deficits in hippocampal plasticity. Here, we examined whether changes in δ, θ, α, β and γ oscillations, or θ-γ coupling accompany the chronic loss of LTP that is observed in the MK801-animal model of psychosis. One and 4 weeks after acute systemic treatment of adult rats with MK801, a potent loss of hippocampal *in vivo* LTP was evident compared to vehicle-treated controls. Overall, the typical pattern of θ-γ oscillations that are characteristic for the successful induction of LTP was altered. In particular, θ-power was lower and an uncoupling of θ-γ oscillations was evident in MK801-treated rats. The alterations in network oscillations that accompany LTP deficits in this animal model may comprise a mechanism through which disturbances in sensory information processing and hippocampal function occur in psychosis. These data suggest that the hippocampus is likely to comprise a very early locus of functional change after instigation of a first-episode psychosis-like state in rodents.

## Introduction

In the hippocampus, information processing and storage are supported by neuronal oscillations Buzsáki and Draguhn, 2004; Dragoi and Buzsáki, 2006), particularly at theta (θ, 4–10 Hz) and gamma (γ, 30–100 Hz) frequencies (Csicsvari et al., 2003; Lubenov and Siapas, 2009; Patel et al., 2012; Buzsáki and Moser, 2013; Zylla et al., 2013) that occur during hippocampus-dependent learning events (Buzsáki and Draguhn, 2004; Tort et al., 2009). Theta and gamma oscillations are physiologically interlinked (Fuchs et al., 2007; Lubenov and Siapas, 2009), and theta-gamma oscillations typically occur in a pattern such that high theta power is accompanied by low gamma power and vice versa (Vida et al., 2006). Theta-gamma coupling, as a neuronal correlate of a learning process, may comprise a ubiquitous phenomenon in the brain that is not the sole domain of hippocampal information processing (Kendrick et al., 2011). It has been proposed that theta-gamma interplay enables the maintenance of a serial order of distinct packages of information (Lisman, 2005) and coupling between theta and low gamma oscillations correlate to the processing of episodic memories (Shirvalkar et al., 2010).

Long-term synaptic information storage that occurs as a consequence of learning is enabled by hippocampal synaptic plasticity (Kemp and Manahan-Vaughan, 2007, 2008). In recent years it has become apparent that hippocampal neuronal oscillations in the theta-gamma frequency ranges are tightly interlinked to synaptic plasticity: on the one hand, long-term potentiation (LTP) triggers reorganization of neural networks in the brain (Canals et al., 2009), on the other hand, a very particular pattern of change in theta-gamma coupling must occur during patterned stimulation of afferent fibers to the hippocampus, in order for persistent (>24 h) LTP to successfully occur (Bikbaev and Manahan-Vaughan, 2007, 2008). In line with this, interventions that prevent persistent (>24 h) LTP from occuring also prevent the characteristic profile of theta-gamma oscillations that typically herald the manifestation of LTP (Bikbaev and Manahan-Vaughan, 2016). Moreover, the deficits in hippocampal LTP that occur in an animal model of Alzheimer’s disease are also accompanied by a loss of this characteristic profile of theta-gamma power and coupling changes (Kalweit et al., 2015). This suggests that alterations in network oscillations that typically accompany the induction of hippocampal synaptic plasticity might be an intrinsic component of, and mechanism underlying, dysfunctional synaptic plasticity and the information storage that it enables.

In the present study, we explored whether deficits in hippocampal function that emerge very early after the initiation of a psychosis-like state in rodents (Wöhrl et al., 2007; Manahan-Vaughan et al., 2008a) are also accompanied by changes in theta-gamma power and coupling: we used an animal model of psychosis that involves treatment with an irreversible antagonist of the glutamatergic N-methyl-D-aspartate receptor (NMDAR) and permits exploration of the immediate response of the hippocampus to the first putative manifestation of psychosis (Wiescholleck and Manahan-Vaughan, 2013a). We focused on neuronal oscillations in the hippocampus because although a variety of cognitive structures in the brain are affected by psychosis, the hippocampus appears to be a very important locus of the disease (Cirillo and Seidman, 2003; Harrison, 2004; Velakoulis et al., 2006; Adriano et al., 2012; Harvey and Se Keefe, 2012).

After the initial manifestation of psychosis (the first-episode), treatment in a time-window of roughly 60 days improves the therapeutic prognosis (Drake et al., 2000; Alvarez-Jimenez et al., 2011). It has been shown that *long-term* changes in the glutamatergic, GABAergic and dopaminergic systems accompany psychosis-like and subsequent schizophrenia-like states in rodents (Seeman et al., 1987; Javitt and Zukin, 1991; Krystal et al., 1994; Gordon et al., 2010; Wiescholleck and Manahan-Vaughan, 2013a). Changes in NMDAR expression and/or function are believed to comprise a fundamental component of the pathophysiology of psychosis (Krystal et al., 1994; Lahti et al., 2001; Coyle et al., 2003; Lindsley et al., 2006; Vrajová et al., 2010; Wiescholleck and Manahan-Vaughan, 2013a), whereby the NMDAR, in turn, is a pivotal element for the successful information encoding in the hippocampus (Morris, 2013). Furthermore NMDAR hypofunction in psychosis (Snyder and Gao, 2013; Steiner et al., 2013), that is also reflected in animal models (Rujescu et al., 2006) may contribute to changes in brain network activity that are associated with the disease (Dawson et al., 2014). Within the hippocampus, structural (Kolomeets et al., 2007), anatomical (Heckers and Konradi, 2002), and biochemical changes (Harrison, 2004; Yin et al., 2012) also progressively develop in psychosis. However, very little is known about the *immediate* changes that are initiated within the abovementioned 60 day therapeutic time-window, after first-episode psychosis, that serve to perpetuate the disorder. In a rodent model of psychosis, we previously observed that initiation of a “first episode”-like behavioral state resulted in an immediate and chronic loss of the ability of the hippocampus to express LTP (Manahan-Vaughan et al., 2008a). Hippocampus-dependent learning was also impaired (Manahan-Vaughan et al., 2008a,b; Wiescholleck and Manahan-Vaughan, 2012, 2013b), suggesting that the hippocampus is a brain structure that may undergo substantial functional changes immediate after the first episode of psychosis. In the present study, we observed that the characteristic profile of theta-gamma power and coupling that accompanies the successful induction of robust LTP in healthy animals (Bikbaev and Manahan-Vaughan, 2007, 2008) is disrupted in this animal model of psychosis. These deficits were sustained long after the occurrence of the “first episode”-like response. These findings suggest that a rapid deterioration of the effectivity of information storage-related theta-gamma activity occurs in the NMDAR-animal model of first-episode psychosis that is associated with a profound loss of hippocampal LTP. These changes may comprise the cellular basis for the disruption of hippocampus-dependent cognition that is known to occur in psychosis. They also suggest that the hippocampus is a potential target for early interventions for the treatment of this disease.

## Materials and Methods

The study was carried out in accordance with the European Communities Council Directive of 22 September, 2010 (2010/63/EU) for care of laboratory animals and after approval of the local ethic committee (Landesamt für Naturschutz, Umweltschutz und Verbraucherschutz, Nordrhein Westfalen, Germany). All efforts were made to minimize the number of animals used.

Nine male Wistar rats (Charles River, Germany, 7–8 weeks at the time of electrode implantation) were used in this study. The animals were housed a humidity- and temperature-controlled Scantainer with a constant 12-h light/dark cycle (lights on from 7 AM to 7 PM). Access to food and water was provided *ad libitum*.

### Electrode Implantation and Recordings of Evoked Potentials

Anesthesia was applied by using sodium pentobarbital (52 mg/kg, intraperitoneally, i.p.). Then the animals underwent stereotaxic chronic implantation of electrodes in the right hemisphere, as described previously (Bikbaev and Manahan-Vaughan, 2007). A bipolar stimulation electrode (polyurethane-coated stainless steel wire, 0.1 mm diameter) was implanted in the medial perforant pathway (6.9 mm posterior to bregma, 4.1 mm lateral to the midline) and a monopolar recording electrode was implanted in the granule cell layer of the dentate gyrus (DG; 3.1 mm posterior to bregma, 1.9 mm lateral to the midline). Following recovery from surgery (7–10 days later), evoked potentials were assessed to verify that they showed the characteristic response profile expected from the medial perforant path-DG input (Aksel-Aksoy and Manahan-Vaughan, 2013). After the conclusion of experiments, histological verification of electrode positions was also conducted. Brain sections (16 μm) were embedded in paraffin, stained according to the Nissl method, using 1% toluidine blue, and then examined using a light microscope. Data from brains in which incorrect electrode localizations or hippocampal misconfigurations were found were excluded from analysis.

**Figure 1:**
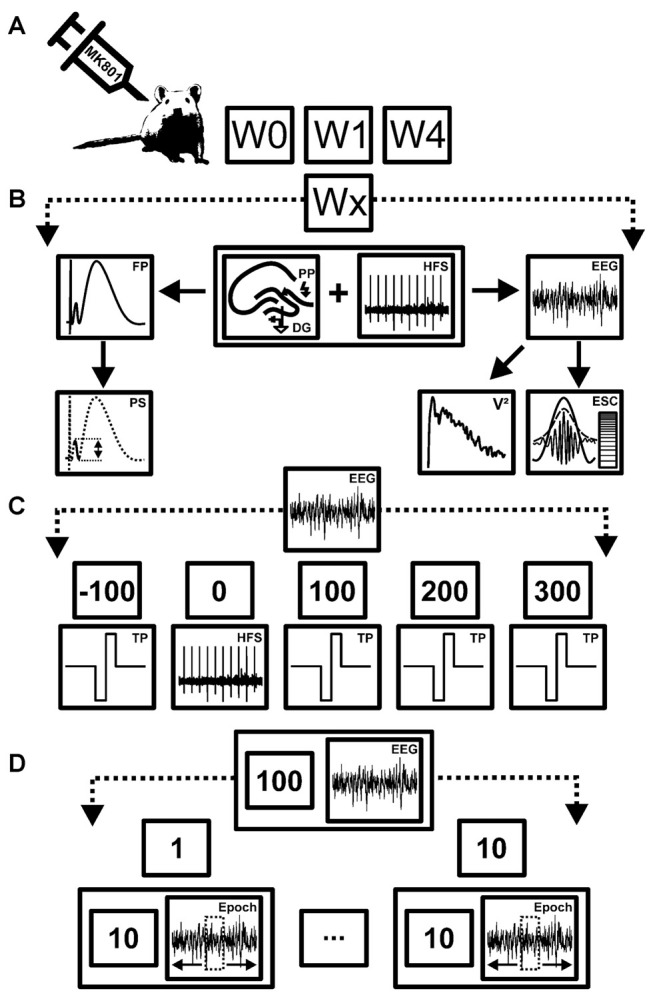
**Overview of treatment, data acquisition and analysis strategy. (A)** Schema of the time-line for treatments and recordings. Animals were first assessed for long-term potentiation (LTP) in the absence of any manipulation (W0). After evoked potentials had returned to pre-LTP levels (7–10 days later), animals were treated with MK801. One week (W1) and 4 weeks after treatment (W4) they were tested for LTP and electroencephalographic (EEG) recordings were made. **(B)** Schema of the stimulation and analysis procedure. During each test condition (Wx) high frequency stimulation (HFS) was applied to the perforant path synapses (PP) to induce LTP in the dentate gyrus (DG). Field potentials (FP) and EEG recordings were obtained concurrently. Both the population spike (PS) and field excitatory postsynaptic potential (fEPSP) were assessed to determine the occurrence and stability of LTP in each rat. EEG measurements from the DG were used to obtain relative power values (V^2^) of neuronal oscillations, as well as envelope-to-signal correlations (ESC) of theta and gamma oscillations. **(C)** Schema of EEG recording strategy. EEG recorded before, during and after HFS, were subdivided into five time windows comprising baseline recordings (*t* = −100 s) prior to HFS, recordings *during* HFS induction (*t* = 0), and *t* = 100 ms, 200 ms and 300 ms after HFS. EEG was recorded for a total of 100 s for each time-point. During continuous EEG recordings biphasic rectangle test-pulses (TP) were applied to evoke FPs. **(D)** Schema of EEG analysis strategy. Each 100 s period of EEG recordings was split into 10 epochs of 10 s time windows each. From each of these 10 s long epochs, one 4.1 s artifact-free epoch was extracted and subjected to analysis. This was done to ensure that stimulation artifacts (caused by TP or HFS) did not contaminate the EEG recordings.

To ensure full habituation to the recording environment, all animals used in the experiments were transferred to the recording chambers (40 (L) × 40 (W) × 40 (H) cm), 1 day before experiments began. The animals could move freely within the recording chambers and had access to food and water *ad libitum*. Field potentials (FP) were evoked by stimulation of the perforant path with test-pulses (TP; 0.025 Hz, single biphasic square wave pulses of 0.2 ms duration). Both the population spike (PS) and the field excitatory postsynaptic potential (fEPSP) were assessed (Bikbaev and Manahan-Vaughan, 2007; Figures 1A,B). Potentials were evoked with a stimulation intensity that elicited a response that comprised 40% of the maximum response observed during an input-output (i/o) assessment (stimulation intensities of 100 μA through 900 μA in increments of to 100 μA) that was conducted prior to each experiment.

For each time-point measured during the LTP experiments, five records of evoked responses were averaged. The first 30 min recorded (six time-points recorded at 5 min intervals) served as baseline reference. All time-points were then calculated as a percentage of the average of the baseline recording. After baseline recordings concluded, high-frequency stimulation (HFS, 15 pulses at 200 Hz, repeated 10 times at 10 s intervals) was applied to elicit LTP. The animals were stationary, resting and had their eyes open at the time-point of HFS. The first three time points after HFS were recorded at 5 min intervals, following by recordings at 15 min intervals for 4 h and for one additional hour 24–25 h after HFS. This protocol is effective in inducing LTP (>24 h) in the DG *in vivo* (Naie and Manahan-Vaughan, 2004; Kemp and Manahan-Vaughan, 2008).

### Neuronal Oscillations

Intra-hippocampal electroencephalogram (EEG) data were obtained during recordings of evoked potentials from the DG granule cell layer, as described previously (Bikbaev and Manahan-Vaughan, 2007; Kalweit et al., 2015; Figure 1C). EEG was recorded using Spike2 software (Cambridge Electronic Design, UK) and stored for subsequent offline analysis. The raw data were sampled at 2 kHz with a 100× gain and were subjected to band-pass filtering at 0.1 Hz–20 kHz. The EEG signal was digitally down-sampled to 1 kHz or 200 Hz, depending on the band-pass filter applied. Large spike artifacts could be seen in the EEG of both vehicle-treated and MK801-treated animals during HFS that corresponded to the 200 Hz stimulation protocol used. This could be used as an orientation to choose the appropriate epochs for EEG analysis.

We assessed five specific time-points: pre-HFS (*t* = −100 ms), *during* HFS (*t* = 0), as well as 100 ms, 200 ms and 300 ms after the conclusion of HFS (Figure 1C). At each time-point 100 s of EEG was recorded. Because HFS involved giving a tetanus at 10 s intervals, we separated the 100 s recordings into 10 epochs of 10 s, and within these epochs selected (one) 4.1 s continuous epochs of artifact-free EEG (Figure 1D). This allowed us to exclude either HFS-related or test-pulse stimulation-related stimulus artifacts from the EEG recordings that were subsequently analyzed.

We assessed oscillatory activity in range of delta (2–4 Hz), theta (4–10 Hz), alpha (10–12 Hz) beta (12–28 Hz) and gamma (30–100 Hz) band activity. A digital infinite impulse response notch-filter was applied to the extracted epochs (−3 dB points for band-stop: 48.5–55 Hz) to remove possible alternating current (AC) from the EEG (Bikbaev and Manahan-Vaughan, 2007; Tsanov and Manahan-Vaughan, 2009; Kalweit et al., 2015). “Baseline” EEG data were obtained in the 100 s recording period prior to HFS, and were used as a reference for analysis of data obtained during or after HFS (i.e., taken as a 100% reference value).

We examined for possible changes in power levels of distinct oscillators by transforming the EEG data using fast Fourier analysis (FFT) function (NFFT = 2048). The mean values for each power spectrum was then calculated by using inbuilt MATLAB functions (MathWorks, Natick, MA, USA, version: R2015b). The absolute values of the spectral power of the artifact-free epochs were determined with respect to the mean values of the 100 s-long baseline period obtained from each individual animal.

We assessed inter-frequency phase-to-amplitude coupling (PAC) to determine envelope-to-signal correlations (ESC) between theta and gamma oscillations using a combination of published Matlab-functions (Onslow et al., 2011) and self-written Matlab-functions (Kalweit et al., 2015). We used the ESC rating (Bruns and Eckhorn, 2004) because of its reliability with regard to short time-series epochs of EEG as these were the focus of our study. We used the following settings for PAC-ESC measurements: morlet wavelet filter width: 7, FFT-size: 200, shuffling windows: 200. Raw wave signal sampling rate: 2 kHz. If not otherwise mentioned, the default settings of the provided function were used. The mean PAC-ESC score for all epochs was acquired for all animals for each test group, specific to the time-windows assessed before, during and after HFS. Later the resulting PAC-ESC score were set in relation to baseline recordings and statistically analyzed.

### MK801 Treatment

In previous studies (Wöhrl et al., 2007; Wiescholleck and Manahan-Vaughan, 2013b), we showed that in the 4 week time-course of the study LTP properties and fEPSP profiles remained unchanged in vehicle-treated animals. Here, in order to be able to closely scrutinize any possible changes in neuronal oscilaltions as a result of MK801-treatment the animals served as their own controls: first, LTP was assessed in the animal cohort (*n* = 9) in the absence of any treatment. Roughly 1 week later, when evoked potentials had returned to pre-HFS levels, the NMDAR antagonist, [+]-5-methyl-10,11-dihydro-5H dibenzo-[a,d]-cyclohepten-5,10-imine hydrogen maleate (MK801, Tocris, Bristol, UK) was applied (5 mg/kg, intraperitoneally, i.p.), as previously described (Manahan-Vaughan et al., 2008a). One week and 4 weeks after MK801 treatment, the ability of the animals to express LTP was assessed (Figures 1A,B).

### Statistical Analysis

For LTP experiments, results were expressed as the mean percentage ± standard error of the mean (SEM) of the average of the first six recordings. Statistical analysis was performed by using inbuilt MATLAB functions (MathWorks, Natick, MA, USA, version: R2015b). The Anderson-Darling-test was applied to each single distribution to assess for normal distribution. If the Anderson-Darling-test was significant, the non-parametric Wilcoxon-test Kruskal-Wallis test and Tukey *post hoc*-test were applied. Otherwise parametric statistical tests (Student’s *t*-test or multifactorial analysis of variance (ANOVA) with repeated measures) were applied. The effect of time included five levels comprising pre-HFS (100 s), during HFS (100 s) and 100 s each of recordings *t* = 100 s, *t* = 200 s and *t* = 300 s post-HFS (Figure 1C). Data were expressed as mean % pre-HFS values ± SEM. The significance level was set at *p* < 0.05 (*).

## Results

### LTP in the Dentate Gyrus of Adult Rats Is Still Impaired Weeks after MK801-Treatment

**Figure 2:**
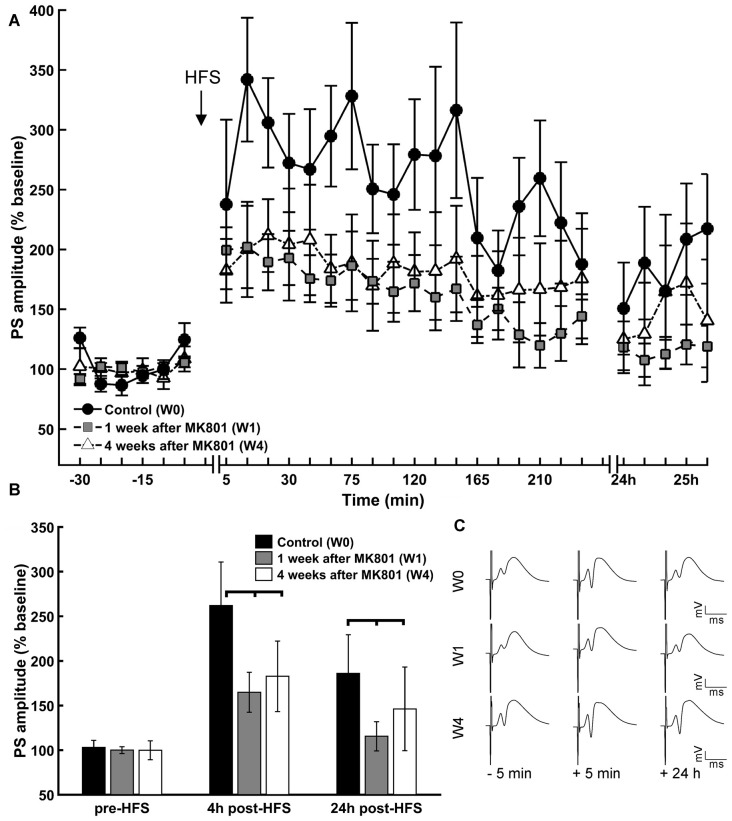
**LTP is impaired 1 and 4 weeks after MK801-treatment. (A)** HFS of the perforant path resulted in LTP in the DG that persisted for over 24 h in untreated animals (W0). One (W1) and 4 weeks (W4) after MK801-treatment LTP was significantly impaired. **(B)** Basal synaptic transmission (pre-HFS) was unaffected by MK801 treatment, but 4 h and 24 h after HFS a significant reduction in potentiation was evident in both the W1 and W4 treatment groups. **(C)** Analog examples of evoked responses obtained 5 min prior to HFS, 5 min and 24 h after HFS in the absence of MK801 treatment (W0), and 1 week (W1) and 4 weeks (W4) after MK801 treatment. Vertical scale bar corresponds to 5 mV; horizontal scale bar corresponds to 5 ms.

Prior to MK801 treatment all animals responded to HFS with robust LTP that lasted for at least 24 h (Figures 2A,C). One week and 4 weeks after treatment with MK801, HFS resulted in LTP that was significantly impaired compared to vehicle-treated rats (Figures 2A–C). This is in line with reports of previous studies (Manahan-Vaughan et al., 2008a).

Responses that were elicited by test-pulse stimulation remained equivalent across the treatment between groups (Figure 2B, ANOVA: *F*_(2)_ = 0.14, *p* > 0.05), suggesting that MK801-treatment did not impact directly on basal synaptic transmission, but 4 h after HFS, potentiation was significantly reduced in both the 1 week and 4 week treatment groups compared to the control *condition* (Figure 2B; $\chi_{\left(2\right)}^{2}$ = 48.21, *p* < 0.01, Tukey-based *post hoc*-analysis: week 0/week 1: *p* < 0.01; week 0/week 4: *p* < 0.01). The same is true for the 24 h *measurement* ($\chi_{\left(2\right)}^{2}$ = 8.58, *p* < 0.05, Tukey-based *post hoc*-analysis: week 0/week 1: *p* < 0.05; week 0/week 4: *p* < 0.05). Twenty-four hours after HFS, potentiation was significantly reduced in both treatment groups compared to the control *condition* (Figure 2B; Tukey *post hoc*-test: $\chi_{\left(2\right)}^{2}$ = 8.58, *p* < 0.05 for control vs. 1 week and control vs. 4 weeks).

Within-group analysis revealed that whereas LTP was sustained for over 24 h in animals prior to treatment, 1 and 4 weeks after MK801 treatment, LTP was sustained for roughly 4 h and completely absent 24 h after HFS (week 0: *F*_(2)_ = 26.53, *p* < 0.01; Tukey-based *post hoc*-analysis: base/4 h: *p* < 0.01, base/24 h: *p* < 0.01, 4 h/24 h: *p* < 0.01; week 1: $\chi_{\left(2\right)}^{2}$ = 50.92 *p* < 0.01; Tukey-based *post hoc*-analysis: base/4 h: *p* < 0.01, base/24 h: *p* > 0.05, 4 h/24 h: *p* < 0.01; week 4: $\chi_{\left(2\right)}^{2}$ = 32.62 *p* < 0.01; Tukey-based *post hoc*-analysis: base/4 h: *p* < 0.01, base/24 h: *p* > 0.05, 4 h/24 h: *p* < 0.01).

### MK801-Treatment Attenuates Hippocampal Network Activity during HFS

**Figure 3:**
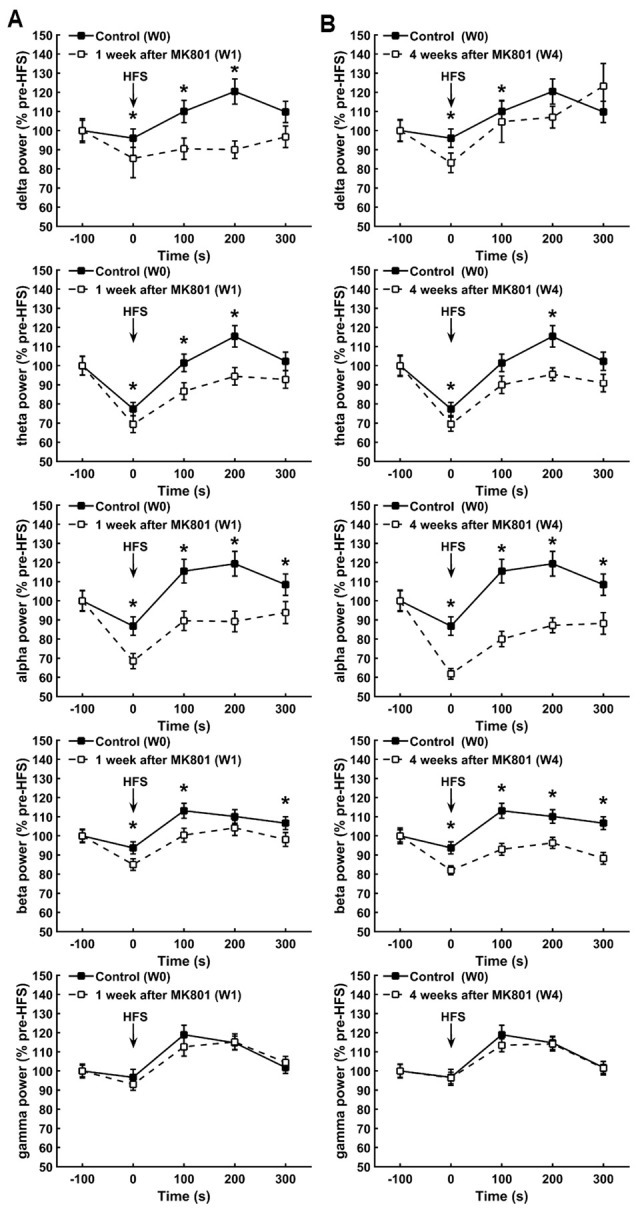
**MK801-treatment disrupts hippocampal network activity during HFS. (A,B)** One **(A)** and 4 weeks **(B)** after MK8010-treatment, significant reductions in the power of delta, theta, alpha, beta but not gamma frequency oscillations were detected during and after HFS compared to responses recorded in the same animals prior to MK801-treatment (*p < 0.05). Values are expressed as a percentage of the pre-HFS baseline obtained prior to HFS in each respective test condition.

Both 1 and 4 weeks after MK801 treatment, oscillations in the delta, theta, alpha and beta frequency band ranges all exhibited significantly lower power values compared to responses obtained during HFS prior to MK801-treatment (Figures 3A,B). One week after MK801 treatment, this suppression of power was sustained for 200 ms after HFS with regard to delta and theta power (between-group, *p* < 0.05) and for 300 ms post-HFS with regard to alpha and beta power, even though effects were relatively small (between-group, *p* < 0.05). Four weeks after MK801-treatment the suppression was still evident 100 ms after HFS with regard to delta power, were sustained for 200 ms after HFS for theta power, and lasted for at least 300 ms post-HFS with regard to beta and alpha power (between-group, *p* < 0.05).

Interestingly, gamma power was not affected by the treatment, where by responses recorded during and after HFS 1 and 4 weeks after treatment remained equivalent to control responses (Figures 3A,B; between group, *p* < 0.05).

### Theta Oscillations during HFS Are Altered by MK801-Treatment

**Figure 4:**
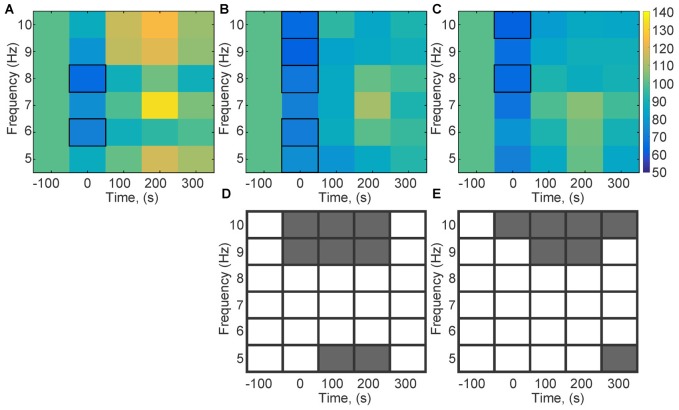
**Theta frequencies during HFS are altered by MK801-treatment.** Changes in theta frequency bands (5, 6, 7, 8, 9, 10 Hz) were assessed during and after HFS. **(A–C)** Values are expressed in the form of a color scale that corresponds to the relative power of the frequency expressed as a percentage of the pre-HFS (baseline) power values of that respective frequency. Color scale values are shown in the bar at the right-hand side of part **(C)**. In the absence of MK801-treatment **(A)**, HFS (*t* = 0) was associated with a significant reduction of theta power across all theta frequency bands. A significant reduction in theta power for all frequency bands was evident during HFS, 1 week **(B)** and 4 weeks **(C)** after MK801 treatment. **(D,C)** Statistical comparisons of theta oscillations (between group comparisons) recorded during, and 100, 200 and 300 ms after HFS, 1 and 4 weeks after MK801 treatment with equivalent frequency bands obtained in the absence of MK801-treatment reveals that 1 week after MK801-treatment **(D)** a significant reduction of 9 and 10 Hz theta frequencies occurs during and for up to 200 s after HFS. Significant reductions in the 5 Hz range occur 100 and 200 s after HFS. Four weeks after MK801-treatment **(E)** reductions in 9 and 10 Hz theta frequencies occur during and for up to 300 s after HFS. A reduction in the 5 Hz range occurs 300 ms after HFS. Gray boxes correspond to a significant difference (between group comparisons) for a specific frequency and time-point. White boxes signify an absence of significant difference.

Theta activity during HFS predicts whether LTP will result from the afferent stimulation (Bikbaev and Manahan-Vaughan, 2007, 2008; Kalweit et al., 2015). Therefore, we scrutinized individual frequencies within the theta range (5, 6, 7, 8, 9 and 10 Hz) to clarify if the response of a specific frequency range to HFS was affected by MK801-treatment (Figure 4). Control animals exhibited a significant reduction in theta amplitude across some theta frequency bands *during* HFS (compared to pre-HFS values (Figure 4A; 8 Hz; $\chi_{\left(4\right)}^{2}$ = 14.8, *p* < 0.05; 6 Hz: $\chi_{\left(4\right)}^{2}$ = 10.95, *p* < 0.05). This response profile was also evident during HFS in animals that had been treated with MK801 1 (Figure 4B) or 4 weeks (Figure 4C) previously, whereby no significant difference was evident when these test conditions were compared to the control *condition* (week 1: 10 Hz; $\chi_{\left(4\right)}^{2}$ = 17.77, *p* < 0.05; 9 Hz; $\chi_{\left(4\right)}^{2}$ = 20.93, *p* < 0.05; 8 Hz; $\chi_{\left(4\right)}^{2}$ = 19.63, *p* < 0.05; 6 Hz; $\chi_{\left(4\right)}^{2}$ = 10.9, *p* < 0.05; 5 Hz; $\chi_{\left(4\right)}^{2}$ = 10.6, *p* < 0.05; week 4: 10 Hz; $\chi_{\left(4\right)}^{2}$ = 20.47; 8 Hz; $\chi_{\left(4\right)}^{2}$ = 17.68). A closer examination of the theta response profiles in MK801-treated animals revealed significantly lower responses in the 9–10 Hz bands during HFS compared to the control HFS condition, however (Figures 4D,E). These reductions were sustained 100 ms and 200 ms after HFS 1 week after treatment (between group test, *p* < 0.05) and for at least 300 ms after HFS in the 4 week treatment condition (between group, *p* < 0.05). Theta power in the 5 Hz range was also significantly suppressed during and for 100 ms after HFS in the 1 week treatment group (between group, *p* < 0.05), whereas a reduction in 5 Hz power also occurred 300 ms after HFS in the 4 week group (between group, *p* < 0.05; Figures 4D,E).

### Phase-to-Amplitude Coupling Is Altered during, but Not After HFS as a Consequence of MK801-Treatment

**Figure 5:**
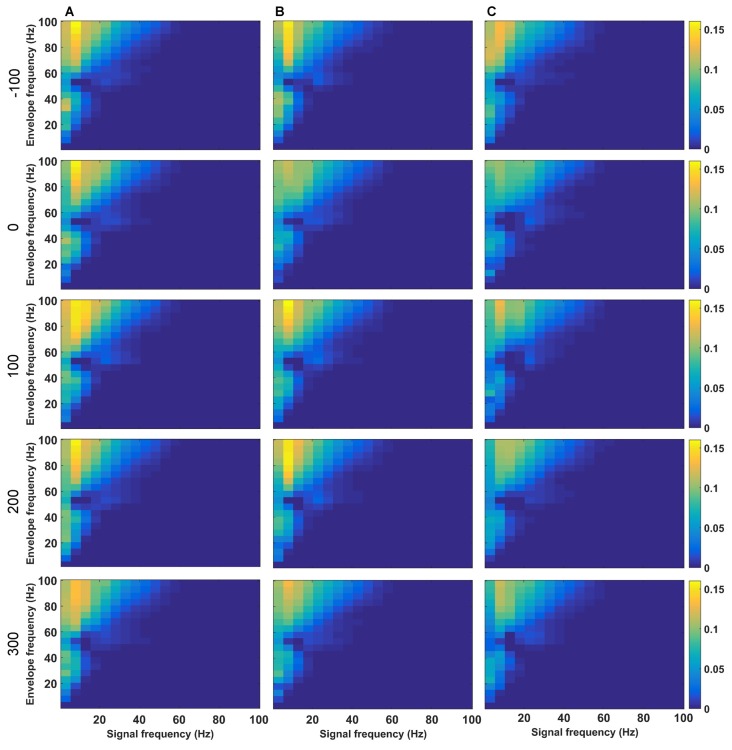
**Phase-to-amplitude coupling (PAC) is altered during, but not after HFS as a consequence of MK801-treatment. (A–C)** Changes in theta coupling as a result of HFS was assessed by determining the PAC-ESC scores in the absence of MK801 **(A)**, and 1 **(B)** or 4 weeks **(C)** after MK801-treatment. Examples show (top-to-bottom) scores obtained pre-HFS (*t* = −100 s), during HFS (*t* = 0), 100 s after HFS, 200 s after HFS and 300 s after HFS. The raw EEG was binned into center frequencies in the range of 3–98 Hz ± 2 Hz resulting in 20 bins in increments of 5 Hz. The *y*-axis reflects the envelopes of the binned signal whereas the *x*-axis reflects the frequencies of the signal amplitudes. The PAC-ESC scores, resulting from a comparison of envelopes and amplitudes at the binned frequencies, are expresses on the color scale for each separate plot. The probability that envelopes of a fast signal match to the shape of amplitudes from slower signals is higher. For this reason the highest PAC-ESC scores are observed at the top left of each single plot. This relationship remains stable for all test-conditions **(A–C)** and time-points. The PAC-ESC scores for frequencies in the range of theta to beta, as well as high gamma oscillation remain high during all time-points and conditions, with the exception of *t* = 0 (during HFS), whereby lower PAC-ESC scores are evident 1 and 4 weeks after MK801-treatment, compared to the control condition.

**Figure 6:**
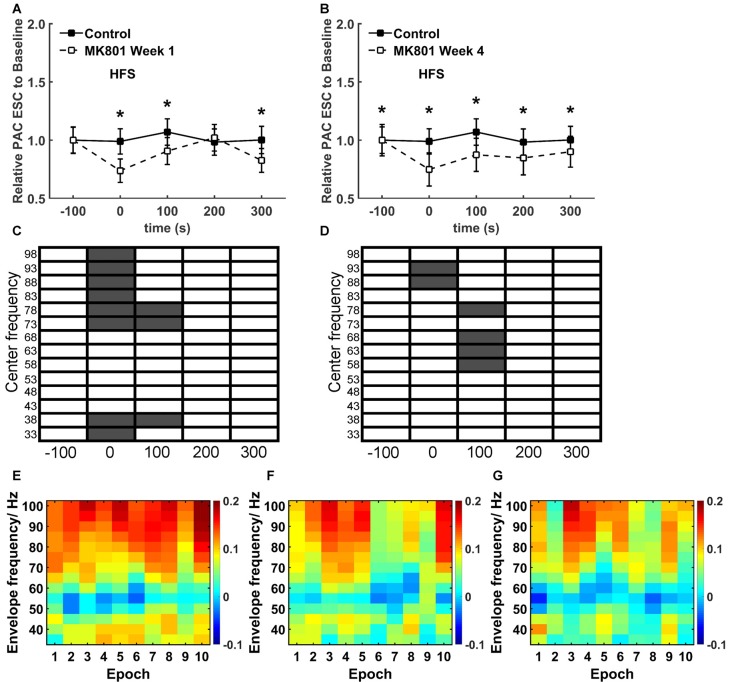
**MK801 treatment elicits specific temporal effects on theta-gamma coupling during HFS. (A,B)** A closer examination of the PAC-ESC scores for theta and gamma oscillations revealed significant reductions (*p < 0.05) during and after HFS both 1 week **(A)** and 4 weeks **(B)** after MK801 treatment, compared to the control condition. Effects were more pronounced 4 weeks after treatment. (Theta-gamma coupling scores for distinct gamma bands relative to theta bands were pooled for this assessment). **(C,D)** When the raw EEG was segregated into center frequencies in the range of 33–98 Hz ± 2 Hz (5 Hz bins), significantly significant reductions in the PAC-ESC scores during HFS (in the range of 73–100Hz) and 100 s after HFS (in the range of 33–17 and 73–82 Hz) were observed 1 week after MK801 treatment **(C)** compared to values obtained in the control condition. Four weeks after MK801-treatment **(D)**, PAC-ESC scores were reduced during HFS (in the range of 88–97 Hz) and reduced 100 s after HFS (in the range of 58–72 and 73–82 Hz) compared to values obtained in the control condition. Gray boxes correspond to a significant difference (Wilcoxon test) for a specific frequency and time-point. White boxes signify an absence of significant difference. **(E–F)** A closer examination of theta-gamma coupling during HFS revealed that whereas coupling remained high throughout the entire HFS period in the control condition **(E)**, 1 week **(F)** and 4 weeks **(G)** after MK801-treatment reductions in theta-gamma coupling emerged during the second half of the 100 s period during which HFS was applied. The charts show the envelope frequency during the 10 separate consecutive epochs that were assessed during the 100 s period of HFS application. The color scale at the right side of part **(G)** represents the theta-gamma coupling scores shown in parts **(E–G)**.

Successful LTP is associated with a very specific profile of theta-gamma coupling during HFS (Bikbaev and Manahan-Vaughan, 2007; Kalweit et al., 2015). Given that high theta frequencies during HFS were altered by MK801-treatment, we examined PAC by determining the ESC for all HFS conditions (Figures 5, 6). PAC-ESC scores obtained for delta, theta, alpha, beta and gamma bands at the different time-points prior to and after HFS in the control group (Figure 5A), the 1 week condition (Figure 5B) and the 4 week condition (Figure 5C) did not reveal any differences. Specifically, the PAC-ESC scores for frequencies in the range of theta, alpha and beta, as well as high gamma remained high during all time-points and conditions, with the exception of during HFS, whereby lower PAC-ESC scores were detected for these frequency ranges 1 and 4 weeks after MK801-treatment, compared to the control condition (see Figure 5 for statistical comparisons).

### Theta-Gamma Coupling Is Altered during HFS as a Consequence of MK801-Treatment

A specific, more detailed, scrutiny of theta-gamma coupling scores revealed significantly lower coupling scores between theta waves and gamma oscillations *during* HFS both 1 week (Figure 6A) and 4 weeks after MK801-treatment (Figure 6B; between group, *p* < 0.05) compared to the control condition.

An examination of the coupling scores for distinct center frequency bands of gamma oscillations and theta waves also revealed significantly lower PAC-ESC coupling scores during HFS both 1 week (Figure 6C) and 4 weeks (Figure 6D) after treatment. Specifically, lower coupling scores for high gamma center frequencies were evident.

To scrutinize this more closely, we assessed the ten individual recording epochs that were obtained during HFS (Figures 6E–G). Here, compared to the control condition (Figure 6E). We found that theta-gamma coupling is lower in the latter 50 s of HFS both 1 week (Figure 6F) and 4 weeks after MK801-treatment (Figure 6G). Given that we observed that gamma oscillations are not impaired by MK801 treatment (Figure 3), the change in theta-gamma coupling is likely to have derived from the changes in theta activity that we observed during HFS (Figure 4).

## Discussion

In this study, we examined whether deficits in hippocampal LTP that occur in an animal model of psychosis are accompanied by changes in neuronal oscillations and in particular in theta-gamma oscillations that are tightly associated with the successful induction of LTP (Bikbaev and Manahan-Vaughan, 2007, 2008). We examined this in the MK801-animal model of psychosis, as changes in hippocampal synaptic plasticity in this model have been characterized in detail (Wiescholleck and Manahan-Vaughan, 2013a). The particular approach we used, is based on a single systemic administration of the irreversible NMDAR antagonist, MK801, as this is believed to trigger a state in rodents that is equivalent to the first episode of psychosis (Wöhrl et al., 2007; Wiescholleck and Manahan-Vaughan, 2013a) and therefore, offers the opportunity to examine how hippocampal function may be altered, by and in close proximity to, the first symptomatic manifestation of this disorder.

We observed that 1 and 4 weeks after treatment of adult rats with MK801, a potent loss of hippocampal LTP was evident compared to LTP elicited in the same animals prior to treatment. HFS of hippocampal afferents that results in LTP that lasts for very long periods (24 h) in behaving rodents is associated with a very specific increase in theta-gamma coupling, a transient decrease followed by an increase in theta power and an transient increase in the power of gamma oscillations (Bikbaev and Manahan-Vaughan, 2007, 2008). This profile was evident in the control condition in the present study, but MK801 treatment significantly reduced both theta-gamma coupling during, and the characteristic theta response during and after HFS. These findings suggest that hippocampal information processing and/or transfer that is enabled by neuronal oscillations may comprise a fundamental component of early changes in the brain that occur in the immediate temporal window during and after first episode psychosis.

Changes in cortical network activity have been reported in first-episode psychosis in humans based on resting state magnetic resonance imaging (MRI) studies (Gong et al., 2017). Interestingly, a heightened inter-network connectivity within the default mode network (Gong et al., 2017), as well as hyperconnectivity that involves the hippocampus is evident in first-episode psychosis (Cui et al., 2015). By contrast, chronic psychosis appears to be associated with a loss of hippocampal connectivity with default mode-related structures (Samudra et al., 2015). These findings suggest in turn, that first-episode psychosis is associated with initial changes in hippocampal (and cortical) function that may in fact be distinct from changes that accompany the perpetuation of the disease. These early changes may relate to changes in hippocampal excitability: we have observed that in the MK801-animal model of first episode psychosis, loss of hippocampal LTP (Manahan-Vaughan et al., 2008a) and of hippocampus-based cognition (Manahan-Vaughan et al., 2008b; Wiescholleck and Manahan-Vaughan, 2013b) may be mediated by potent elevations in neuronal excitability (Kehrer et al., 2007; Grüter et al., 2015), erroneous neuronal information encoding, disruptions of GABAergic inhibitory control and an uncoupling of hippocampal-prefrontal cortex circuitry (Grüter et al., 2015).

Elevations of hippocampal excitability can be expected to impair LTP by changing the dynamic capacity of the hippocampus to engage in selective potentiations of synaptic strength. Elevating the sensitivity of hippocampal neurons to incoming information, by lowering their depolarization thresholds, can also be expected to impair information discrimination and encoding at the level of pattern completion and/or pattern separation (Hunsaker and Kesner, 2013) by increasing interference and lowering signal-to-noise ratios. Interestingly, a loss of pattern separation ability has been reported in schizophrenic patients that is ascribed to a dysfunction of the DG (Das et al., 2014). In forms of psychosis that are related to schizophrenia, positive symptoms (delusions, hallucinations and thought disorder) and cognitive deficits are evident (Harrison, 1999). The abovementioned findings provoke the question as to whether changes in cognitive function in psychosis relate to specific functional changes in the hippocampus that can be detected at the level of neuronal oscillations.

Neuronal oscillations in different frequency bands reflect different forms of information processing and cognitive activity within the hippocampus. Whereas gamma activity is believed to reflect processes such as temporal encoding (Buzsáki and Chrobak, 1995; Lisman and Idiart, 1995), context association (Gray and Singer, 1989) and both the encoding and retrieval of experiences (Lisman and Idiart, 1995; Jensen and Lisman, 1996), theta activity reflects phenomena such as movement-related exploratory behavior and attentional processes (Vanderwolf, 1969) and may support the encoding of space and time by the hippocampus. Theta and gamma activity are closely interlinked however, and phase-locking of gamma oscillations with theta waves occurs during spatial learning and neuronal encoding of space (Dragoi and Buzsáki, 2006). Delta activity relates to attention to rhythmic patterns of sensory stimuli (Lakatos et al., 2005, 2008) that has also been described in the hippocampus (Ringo et al., 1994; Rajkai et al., 2008). Along with theta-gamma coupling, alpha and beta activity are believed to correlate specifically to the encoding and reactivation of episodic memories (Hanslmayr et al., 2016). Abnormal cortical theta and gamma oscillations have been reported in schizophrenia (Kwon et al., 1999; Koukkou et al., 2000; Ford and Mathalon, 2005) that, taken together with reports of changed functional connectivity based on the MRI studies mentioned above, suggest that changes at the level of the hippocampus are not unlikely.

We observed that in an animal model of psychosis, the triggering of a psychosis-like state by treatment with the irreversible NMDAR antagonist, MK801, profoundly impaired the ability of the hippocampus to express LTP. Although basal levels of neuronal oscillations were unchanged in all frequency bands assessed (δ, θ, α, β and γ), application of HFS to the perforant path was not only associated with an impairment of LTP, it was also associated with a suppression of neuronal oscillations in all frequency bands, with the exception of gamma. Beta frequency oscillations were particularly affected. LTP is tightly associated with the encoding of novel space (Kemp and Manahan-Vaughan, 2004, 2007) and an elevation of beta power is related to the perception of environmental novelty in rodents (Berke et al., 2008). We also observed significant reductions in delta and alpha power. Both oscillators have been shown to emerge in the thalamus and are propagated to the hippocampal region (Hughes and Crunelli, 2005; Zhang et al., 2012). Given the role of the thalamus in processing sensory information, the reductions in alpha, delta and beta power may be interrelated. These changes can not only be expected to impact on the effective induction of functionally relevant LTP, they may also contribute to hippocampus-dependent changes in sensory information processing that occur in psychosis (Bast and Feldon, 2003; Sehatpour et al., 2010).

We observed that the higher frequency bands of theta (9–10 Hz) were those most particularly affected by MK801-treatment. Whereas low theta frequencies theta relate to attentional processes that are triggered by septal cholinergic modulation of the hippocampus (Kramis et al., 1975; Buzsáki, 2002), theta frequencies in the range of 7–12 Hz are induced by NMDAR activation (Bland et al., 2007). MK801-treatment results in irreversible binding of the antagonist to the NMDAR that in turn may trigger NMDAR-hypofunction. NMDAR-hypofunction, in turn, is believed to comprise an intrinsic mechanistic change in psychosis (Coyle, 2012; Ju and Cui, 2016). Thus, the reductions in high theta that we observed in the present study, not only provide novel evidence that LTP induction is related specifically to changes in high theta power, they also suggest that changes in NMDAR function may lie at the core of changes in hippocampal theta that occur in psychosis (Cousijn et al., 2015).

An increase in the coupling of theta-gamma oscillations, that is associated with a very particular profile of change in theta and gamma power during and after HFS is a very robust predictor for the successful induction of LTP that lasts for at least 24 h in rodents (Bikbaev and Manahan-Vaughan, 2007, 2008). A poorer change in coupling and profile is associated with the induction of short-term potentiation by the same HFS protocol, whereas an absence of this characteristic change in theta-gamma coupling and power predicts for the complete failure of HFS to elicit any change in synaptic strength at all (Bikbaev and Manahan-Vaughan, 2007, 2008). In the present study, we observed that theta-gamma coupling during HFS was weakened by MK801 treatment, which in turn could explain in part, why LTP is impaired. Interestingly, impairments in HFS-related changes in theta-gamma coupling also occurs in an animal model of Alzheimer’s disease that also display significant deficits in LTP (Kalweit et al., 2015).

Curiously, the deficits in theta-gamma coupling, that we observed, appeared to be determined by the reductions in theta power that were caused by MK801-treatment: we detected no significant changes in gamma power at all. Gamma oscillations are induced by parvalbumin-positive interneurons that respond to theta activity (Fuchs et al., 2007). Thus, an almost inevitable inter-relationship of both frequency bands can be assumed to occur on a physiological level. Considered from this perspective, it is quite perplexing that gamma oscillations were not affected by MK801-treatment. Two possible explanations come to mind: either the gamma band in the range of 30–100 Hz is not functionally connected to the high theta band at all, or a functional disconnection of the high theta band from the gamma band was triggered by the MK801 treatment. An argument against the former option is evident in observations that high theta modulates high gamma related to the speed of physical movement whilst rodents engage in spatial navigation (Chen et al., 2011). This suggests that a dissociation of high theta from gamma may have occurred. Given the assumption that movement-speed related theta-gamma coupling enables adaptation of the learning of temporal sequences to the speed of navigation (Chen et al., 2011), one might expect that a functional consequence of a dissociation of high theta from gamma would comprise an impairment of sequence learning. Interestingly, spatial sequence learning is impaired in first episode psychosis in human subjects (Fajnerová et al., 2014).

It has been reported that neurotoxicity, particularly resulting in cell death, can result from MK801 treatment (Fix et al., 1993). In previous studies, we closely scrutinized whether MK801, given in the precise dose and protocol used in the present study, affects cell viability. We neither found evidence of neurotoxic or necrotic effects in the hippocampus (Wöhrl et al., 2007), nor did we identify any MK801-related cell loss in the entorhinal or retrosplenial cortices (Wiescholleck and Manahan-Vaughan, 2013b). The differences in our observations compared to the study by Fix et al. (1993) may relate to differences in the treatment approach (intraperitoneal vs. subcutaneous application), or the rat strain (Wistar vs. Sprague Dawley). We cannot entirely exclude that extrahippocampal MK801-mediated neurotoxicity contributed in some way to the changes in hippocampal function that we report in the present study, however. The necrosis registered in the retrosplenial cortex using light microscopy in the study by Fix et al. (1993) was further verified using electron microscopy. Although we saw no changes in cell viability using a variety of histological/immuno staining approaches including Hematoxylin and Eosin, Nissl (Toluidine blue) double-staining with Fluoro-Jade B and 4’,6-diamidino-2-phenylindole (DAPI), or immunostaining for Caspase-3 (Wöhrl et al., 2007; Wiescholleck and Manahan-Vaughan, 2013b), we predominantly focussed on the hippocampus.

Although it is clear that changes in multiple neurotransmitter systems, including the dopaminergic (Seeman, 1987), GABAergic (Gordon et al., 2010) and glutamatergic (NMDAR) systems (Moghaddam, 2003; Lindsley et al., 2006; Wiescholleck and Manahan-Vaughan, 2013a) all contribute to the pathophysiology of schizophrenia, as yet, the temporal dynamics of changes in these systems is unclear: do changes in these neurotransmitter systems occur in parallel, or does an imbalance in one system drive changes in the others? Very little is known about changes in neurotransmitter receptor expression during and after first-episode psychosis, but one study that analyzed receptor expression in the blood of first episode patients prior to anti-psychotic treatment reported changes in GABA_A_ gene expression (Ota et al., 2014), suggesting that this receptor undergoes early changes. On the other hand, treatment of rodents with MK801 alters dopamine receptor expression in the hippocampus (Healy and Meador-Woodruff, 1996), changes the firing rates of dopaminergic neurons, and alters GABA receptor expression in the prefrontal cortex (Grüter et al., 2015), a structure that is also intrinsically altered in psychosis (notably also in the context of NMDAR hypofunction in humans (Jodo, 2013). In rodents NMDAR receptor antagonists cause reductions in parvalbumin mRNA (Cochran et al., 2003) and reduce parvalbumin and GAD67 levels in parvalbumin-expressing interneurons (Kinney et al., 2006), suggesting that changing NMDAR activity also alters GABAergic function. In line with this, changes in the regulation by NMDAR of fast-spiking (GABAergic) interneurons is believed to comprise an intrinsic mechanistic component of psychosis (Bitanihirwe et al., 2009; Belforte et al., 2010). The changes in neuronal oscillations that occurred following MK801-treatment were evident 1 week after treatment and sustained for at least 4 weeks. Taken together with other studies that showed that positive modulation of NMDAR or of phosphodiesterase type 4 (PDE4) within weeks of MK801-treatment can restore LTP and spatial learning deficits in this animal model of first-episode psychosis (Manahan-Vaughan et al., 2008b; Wiescholleck and Manahan-Vaughan, 2012), and that changes in neuronal excitability and plasticity-related neurotransmitter receptor expression occurs soon after MK801-treatment (Grüter et al., 2015), this suggests that the hippocampus may lie at the core of early functional changes that contribute to the pathophysiology and perpetuation of schizophrenia.

## Conclusion

This study showed that the typical pattern of theta-gamma oscillations that are characteristic for the successful induction of LTP is chronically altered following MK801-treatment to emulate the manifestation of first-episode psychosis. The power of other frequency bands is also affected, suggesting that a psychosis-like state in rats impacts potently on hippocampus-dependent information processing. Another striking finding of this study is that a rapid deterioration of the effectivity of information storage-related theta-gamma activity becomes apparent within days of the triggering of a first-episode psychosis-like state in rodents. This is associated with a profound loss of hippocampal LTP. These changes may comprise the cellular basis for the disruption of hippocampus-dependent cognition that is known to occur in psychosis. They also suggest that the hippocampus is a potential target for early interventions for the treatment of psychosis.

## Author Contributions

DM-V created the concept and strategy of the study; JC-K conducted the electrophysiological manipulations and experiments; LTP data analysis was conducted by JC-K and DM-V; EEG analysis was conducted by ANK, BA-G and DM-V; the article was written by DM-V with contributions from all authors.

## Conflict of Interest Statement

The authors declare that the research was conducted in the absence of any commercial or financial relationships that could be construed as a potential conflict of interest.

## References

[B1] Adriano F. Caltagirone C. Spalletta G. (2012). Hippocampal volume reduction in first-episode and chronic schizophrenia: a review and meta-analysis. Neuroscientist 18, 180–200. 10.1177/1073858410395147 21531988

[B2] Aksel-Aksoy A. Manahan-Vaughan D. (2013). The temporoammonic input to the hippocampal CA1 region displays distinctly different synaptic plasticity compared to the Schaffer collateral input *in vivo*: significance for synaptic information processing. Front. Synaptic Neurosci. 5:5. 10.3389/fnsyn.2013.00005 23986697 PMC3750210

[B3] Alvarez-Jimenez M. Gleeson J. F. Henry L. P. Harrigan S. M. Harris M. G. Amminger G. P.. (2011). Prediction of a single psychotic episode: a 7.5-year, prospective study in first-episode psychosis. Schizophr. Res. 125, 236–246. 10.1016/j.schres.2010.10.020 21081266

[B4] Bast T. Feldon J. (2003). Hippocampal modulation of sensorimotor processes. Prog. Neurobiol. 70, 319–345. 10.1016/s0301-0082(03)00112-6 12963091

[B5] Belforte J. E. Zsiros V. Sklar E. R. Jiang Z. Yu G. Li Y.. (2010). Postnatal NMDA receptor ablation in corticolimbic interneurons confers schizophrenia-like phenotypes. Nat. Neurosci. 13, 76–83. 10.1038/nn.2447 19915563 PMC2797836

[B6] Berke J. D. Hetrick V. Breck J. Greene R. W. (2008). Transient 23–30 Hz oscillations in mouse hippocampus during exploration of novel environments. Hippocampus 18, 519–529. 10.1002/hipo.20435 18398852

[B101] Bikbaev A. Manahan-Vaughan D. (2016). Metabotropic glutamate receptor, mGlu5, regulates hippocampal synaptic plasticity and is required for tetanisation-triggered changes in theta and gamma oscillations. Neuropharmacology [Epub ahead of print]. 10.1016/j.neuropharm.2016.06.004 27395786

[B7] Bikbaev A. Manahan-Vaughan D. (2007). Hippocampal network activity is transiently altered by induction of long-term potentiation in the dentate gyrus of freely behaving rats. Front. Behav. Neurosci. 1:7. 10.3389/neuro.08.007.2007 18958189 PMC2525854

[B8] Bikbaev A. Manahan-Vaughan D. (2008). Relationship of hippocampal theta and gamma oscillations to potentiation of synaptic transmission. Front. Neurosci. 2, 56–63. 10.3389/neuro.01.010.2008 18982107 PMC2570077

[B9] Bitanihirwe B. K. Y. Lim M. P. Kelley J. F. Kaneko T. Woo T. U. W. (2009). Glutamatergic deficits and parvalbumin-containing inhibitory neurons in the prefrontal cortex in schizophrenia. BMC Psychiatry 9:71. 10.1186/1471-244x-9-71 19917116 PMC2784456

[B10] Bland B. H. Declerck S. Jackson J. Glasgow S. Oddie S. (2007). Septohippocampal properties of N-methyl-D-aspartate-induced theta-band oscillation and synchrony. Synapse 61, 185–197. 10.1002/syn.20357 17173326

[B11] Bruns A. Eckhorn R. (2004). Task-related coupling from high- to low-frequency signals among visual cortical areas in human subdural recordings. Int. J. Psychophysiol. 51, 97–116. 10.1016/j.ijpsycho.2003.07.001 14693360

[B14] Buzsáki G. (2002). Theta oscillations in the hippocampus. Neuron 33, 325–340. 10.1016/s0896-6273(02)00586-x 11832222

[B102] Buzsáki G. Chrobak J. J. (1995). Temporal structure in spatially organized neuronal ensembles: a role for interneuronal networks. Curr. Opin. Neurobiol. 5, 504–510. 10.1016/0959-4388(95)80012-3 7488853

[B12] Buzsáki G. Draguhn A. (2004). Neuronal oscillations in cortical networks. Science 304, 1926–1929. 10.1126/science.1099745 15218136

[B13] Buzsáki G. Moser E. (2013). Memory, navigation and theta rhythm in the hippocampal-entorhinal system. Nat. Neurosci. 16, 130–138. 10.1038/nn.3304 23354386 PMC4079500

[B15] Canals S. Beyerlein M. Merkle H. Logothetis N. K. (2009). Functional MRI evidence for LTP-induced neural network reorganization. Curr. Biol. 19, 398–403. 10.1016/j.cub.2009.01.037 19230667

[B16] Chen Z. Resnik E. McFarland J. M. Sakmann B. Mehta M. R. Borst A. (2011). Speed controls the amplitude and timing of the hippocampal gamma rhythm. PLoS One 6:e21408. 10.1371/journal.pone.0021408 21731735 PMC3123337

[B17] Cirillo M. A. Seidman L. J. (2003). Verbal declarative memory dysfunction in schizophrenia: from clinical assessment to genetics and brain mechanisms. Neuropsychol. Rev. 13, 43–77. 10.1023/A:1023870821631 12887039

[B18] Cochran S. M. Kennedy M. McKerchar C. E. Steward L. J. Pratt J. A. Morris B. J. (2003). Induction of metabolic hypofunction and neurochemical deficits after chronic intermittent exposure to phencyclidine: differential modulation by antipsychotic drugs. Neuropsychopharmacology 28, 265–275. 10.1038/sj.npp.1300031 12589379

[B19] Cousijn H. Tunbridge E. M. Rolinski M. Wallis G. Colclough G. L. Woolrich M. W.. (2015). Modulation of hippocampal theta and hippocampal-prefrontal cortex function by a schizophrenia risk gene. Hum. Brain Mapp. 36, 2387–2395. 10.1002/hbm.22778 25757652 PMC4672713

[B21] Coyle J. T. (2012). NMDA receptor and schizophrenia: a brief history. Schizophr. Bull. 38, 920–926. 10.1093/schbul/sbs076 22987850 PMC3446237

[B20] Coyle J. T. Tsai G. Goff D. (2003). Converging evidence of NMDA receptor hypofunction in the pathophysiology of schizophrenia. Ann. N Y Acad. Sci. 1003, 318–327. 10.1196/annals.1300.020 14684455

[B22] Csicsvari J. Jamieson B. Wise K. D. Buzsáki G. (2003). Mechanisms of gamma oscillations in the hippocampus of the behaving rat. Neuron 37, 311–322. 10.1016/s0896-6273(02)01169-8 12546825

[B23] Cui L. B. Liu J. Wang L. X. Li C. Xi Y. B. Guo F.. (2015). Anterior cingulate cortex-related connectivity in first-episode schizophrenia: a spectral dynamic causal modeling study with functional magnetic resonance imaging. Front. Hum. Neurosci. 9:589. 10.3389/fnhum.2015.00589 26578933 PMC4630283

[B24] Das T. Ivleva E. I. Wagner A. D. Stark C. E. Tamminga C. A. (2014). Loss of pattern separation performance in schizophrenia suggests dentate gyrus dysfunction. Schizophr. Res. 159, 193–197. 10.1016/j.schres.2014.05.006 25176349 PMC4177293

[B25] Dawson N. Xiao X. McDonald M. Higham D. J. Morris B. J. Pratt J. A. (2014). Sustained NMDA receptor hypofunction induces compromised systems integration and schizophrenia-like alterations in functional brain networks. Cereb. Cortex 24, 452–464. 10.1093/cercor/bhs322 23081884

[B26] Dragoi G. Buzsáki G. (2006). Temporal encoding of place sequences by hippocampal cell assemblies. Neuron 50, 145–157. 10.1016/j.neuron.2006.02.023 16600862

[B27] Drake R. J. Haley C. J. Akhtar S. Lewis S. W. (2000). Causes and consequences of duration of untreated psychosis in schizophrenia. Brit. J. Psychiatry 177, 511–515. 10.1192/bjp.177.6.511 11102325

[B103] Fajnerová I. Rodriguez M. Levčík D. Konrádová L. Mikoláš P. Brom C.. (2014). A virtual reality task based on animal research—spatial learning and memory in patients after the first episode of schizophrenia. Front. Behav. Neurosci. 8:157. 10.3389/fnbeh.2014.00157 24904329 PMC4034703

[B28] Fix A. S. Horn J. W. Wightman K. A. Johnson C. A. Long G. G. Storts R. W.. (1993). Neuronal vacuolization and necrosis induced by the noncompetitive N-methyl-D-aspartate (NMDA) antagonist MK(+)801 (dizocilpine maleate): a light and electron microscopic evaluation of the rat retrosplenial cortex. Exp. Neurol. 123, 204–215. 10.1006/exnr.1993.1153 8405286

[B29] Ford J. M. Mathalon D. H. (2005). Corollary discharge dysfunction in schizophrenia: can it explain auditory hallucinations? Int. J. Psychophysiol. 58, 179–189. 10.1016/j.ijpsycho.2005.01.014 16137779

[B30] Fuchs E. C. Zivkovic A. R. Cunningham M. O. Middleton S. L. Lebeau F. E. Bannerman D. M.. (2007). Recruitment of parvalbumin-positive interneurons determines hippocampal function and associated behavior. Neuron 53, 591–604. 10.1016/j.neuron.2007.01.031 17296559

[B31] Gong Q. Hu X. Pettersson-Yeo W. Xu X. Lui S. Crossley N.. (2017). Network-level dysconnectivity in drug-naïve first episode psychosis: dissociating trans-diagnostic and diagnosis-specific alterations. Neuropsychopharmacology 42, 933–940. 10.1038/npp.2016.247 27782128 PMC5312071

[B32] Gordon E. Palmer D. M. Cooper N. (2010). EEG alpha asymmetry in schizophrenia, depression, PTSD, panic disorder, ADHD and conduct disorder. Clin. EEG. Neurosci. 41, 178–183. 10.1177/155005941004100404 21077569

[B104] Gray C. M. Singer W. (1989). Stimulus-specific neuronal oscillations in orientation columns of cat visual cortex. Proc. Natl. Acad. Sci. U S A 86, 1698–1702. 10.1073/pnas.86.5.1698 2922407 PMC286768

[B33] Grüter T. Wiescholleck V. Dubovyk V. Aliane V. Manahan-Vaughan D. (2015). Altered neuronal excitability underlies impaired hippocampal function in an animal model of psychosis. Front. Behav. Neurosci. 9:117. 10.3389/fnbeh.2015.00117 26042007 PMC4438226

[B34] Hanslmayr S. Staresina B. P. Bowman H. (2016). Oscillations and episodic memory: addressing the synchronization/desynchronization conundrum. Trends Neurosci. 39, 16–25. 10.1016/j.tins.2015.11.004 26763659 PMC4819444

[B35] Harrison P. J. (2004). The hippocampus in schizophrenia: a review of the neuropathological evidence and its pathophysiological implications. Psychopharmacology 174, 151–162. 10.1007/s00213-003-1761-y 15205886

[B36] Harrison P. J. (1999). The neuropathology of schizophrenia: a critical review of the data and their interpretation. Brain 122, 593–624. 10.1093/brain/122.4.593 10219775

[B37] Harvey P. D. Se Keefe R. (2012). Technology, society, and mental illness: challenges and opportunities for assessment and treatment. Innov. Clin. Neurosci. 9, 47–50. 23346519 PMC3552462

[B105] Healy D. J. Meador-Woodruff J. H. (1996). Differential regulation, by MK-801, of dopamine receptor gene expression in rat nigrostriatal and mesocorticolimbic systems. Brain Res. 708, 38–44. 10.1016/0006-8993(95)01241-9 8720857

[B39] Heckers S. Konradi C. (2002). Hippocampal neurons in schizophrenia. J. Neural. Transm. 109, 891–905. 10.1007/s007020200073 12111476 PMC4205576

[B40] Hughes S. W. Crunelli V. (2005). Thalamic mechanisms of EEG alpha rhythms and their pathological implications. Neuroscientist 11, 357–372. 10.1177/1073858405277450 16061522

[B41] Hunsaker M. R. Kesner R. P. (2013). The operation of pattern separation and pattern completion processes associated with different attributes or domains of memory. Neurosci. Biobehav. Rev. 37, 36–58. 10.1016/j.neubiorev.2012.09.014 23043857

[B42] Javitt D. C. Zukin S. R. (1991). Recent advances in the phencyclidine model of schizophrenia. Am. J. Psychiatry 148, 1301–1308. 10.1176/ajp.148.10.1301 1654746

[B106] Jensen O. Lisman J. E. (1996). Theta/gamma networks with slow NMDA channels learn sequences and encode episodic memory: role of NMDA channels in recall. Learn. Mem. 3, 264–278. 10.1101/lm.3.2-3.264 10456096

[B43] Jodo E. (2013). The role of the hippocampo-prefrontal cortex system in phencyclidine-induced psychosis: a model for schizophrenia. J. Physiol. Paris 107, 434–440. 10.1016/j.jphysparis.2013.06.002 23792022

[B44] Ju P. Cui D. (2016). The involvement of N-methyl-D-aspartate receptor (NMDAR) subunit NR1 in the pathophysiology of schizophrenia. Acta Biochim. Biophys. Sin. (Shanghai) 48, 209–219. 10.1093/abbs/gmv135 26837414 PMC4885128

[B45] Kalweit A. N. Yang H. Colitti-Klausnitzer J. Fülöp L. Bozsó Z. Penke B.. (2015). Acute intracerebral treatment with amyloid-beta (1–42) alters the profile of neuronal oscillations that accompany LTP induction and results in impaired LTP in freely behaving rats. Front. Behav. Neurosci. 9:103. 10.3389/fnbeh.2015.00103 25999827 PMC4422036

[B46] Kehrer C. Dugladze T. Maziashvili N. Wójtowicz A. Schmitz D. Heinemann U.. (2007). Increased inhibitory input to CA1 pyramidal cells alters hippocampal gamma frequency oscillations in the MK-801 model of acute psychosis. Neurobiol. Dis. 25, 545–552. 10.1016/j.nbd.2006.10.015 17169567

[B47] Kemp A. Manahan-Vaughan D. (2004). Hippocampal long-term depression and long-term potentiation encode different aspects of novelty acquisition. Proc. Natl. Acad. Sci. U S A 101, 8192–8197. 10.1073/pnas.0402650101 15150407 PMC419579

[B48] Kemp A. Manahan-Vaughan D. (2007). Hippocampal long-term depression: master or minion in declarative memory processes? Trends Neurosci. 30, 111–118. 10.1016/j.tins.2007.01.002 17234277

[B49] Kemp A. Manahan-Vaughan D. (2008). The hippocampal CA1 region and dentate gyrus differentiate between environmental and spatial feature encoding through long-term depression. Cereb. Cortex 18, 968–977. 10.1093/cercor/bhm136 17702951

[B50] Kendrick K. M. Zhan Y. Fischer H. Nicol A. U. Zhang X. Feng J. (2011). Learning alters theta amplitude, theta-gamma coupling and neuronal synchronization in inferotemporal cortex. BMC Neurosci. 12:55. 10.1186/1471-2202-12-55 21658251 PMC3123243

[B51] Kinney J. W. Davis C. N. Tabarean I. Conti B. Bartfai T. Behrens M. M. (2006). A specific role for NR2A-containing NMDA receptors in the maintenance of parvalbumin and GAD67 immunoreactivity in cultured interneurons. J. Neurosci. 26, 1604–1615. 10.1523/JNEUROSCI.4722-05.2006 16452684 PMC6675509

[B52] Kolomeets N. S. Orlovskaya D. D. Uranova N. A. (2007). Decreased numerical density of CA3 hippocampal mossy fiber synapses in schizophrenia. Synapse 61, 615–621. 10.1002/syn.20405 17476682

[B53] Koukkou M. Federspiel A. Bräker E. Hug C. Kleinlogel H. Merlo M. C.. (2000). An EEG approach to the neurodevelopmental hypothesis of schizophrenia studying schizophrenics, normal controls and adolescents. J. Psychiatr. Res. 34, 57–73. 10.1016/s0022-3956(99)00040-0 10696833

[B54] Kramis R. Vanderwolf C. H. Bland B. H. (1975). Two types of hippocampal rhythmical slow activity in both the rabbit and the rat: relations to behavior and effects of atropine, diethyl ether, urethane, and pentobarbital. Exp. Neurol. 49, 58–85. 10.1016/0014-4886(75)90195-8 1183532

[B55] Krystal J. H. Karper L. P. Seibyl J. P. Freeman G. K. Delaney R. Bremner J. D. (1994). Subanesthetic effects of the noncompetitive NMDA antagonist, ketamine, in humans. Psychotomimetic, perceptual, cognitive, and neuroendocrine responses. Arch. Gen. Psychiatry 51, 199–214. 10.1001/archpsyc.1994.03950030035004 8122957

[B56] Kwon J. S. O’Donnell B. F. Wallenstein G. V. Greene R. W. Hirayasu Y. Nestor P. G.. (1999). Gamma frequency-range abnormalities to auditory stimulation in schizophrenia. Arch. Gen. Psychiatry 56, 1001–1005. 10.1001/archpsyc.56.11.1001 10565499 PMC2863027

[B57] Lahti A. C. Weiler M. A. Tamara Michaelidis B. A. Parwani A. Tamminga C. A. (2001). Effects of ketamine in normal and schizophrenic volunteers. Neuropsychopharmacology 25, 455–467. 10.1016/s0893-133x(01)00243-3 11557159

[B58] Lakatos P. Karmos G. Mehta A. D. Ulbert I. Schroeder C. E. (2008). Entrainment of neuronal oscillations as a mechanism of attentional selection. Science 320, 110–113. 10.1126/science.1154735 18388295

[B59] Lakatos P. Shah A. S. Knuth K. H. Ulbert I. Karmos G. Schroeder C. E. (2005). An oscillatory hierarchy controlling neuronal excitability and stimulus processing in the auditory cortex. J. Neurophysiol. 94, 1904–1911. 10.1152/jn.00263.2005 15901760

[B61] Lisman J. (2005). The theta/gamma discrete phase code occuring during the hippocampal phase precession may be a more general brain coding scheme. Hippocampus 15, 913–922. 10.1002/hipo.20121 16161035

[B60] Lindsley C. W. Shipe W. D. Wolkenberg S. E. Theberge C. R. Williams D. L. Sur C.. (2006). Progress towards validating the NMDA receptor hypofunction hypothesis of schizophrenia. Curr. Top. Med. Chem. 6, 771–785. 10.2174/156802606777057599 16719816

[B107] Lisman J. E. Idiart M. A. (1995). Storage of 7+/−2 short-term memories in oscillatory subcycles. Science 267, 1512–1515. 10.1126/science.7878473 7878473

[B62] Lubenov E. V. Siapas A. G. (2009). Hippocampal theta oscillations are travelling waves. Nature 459, 534–539. 10.1038/nature08010 19489117

[B63] Manahan-Vaughan D. Haebler D. Winter C. Juckel G. Heinemann U. (2008a). A single application of MK801 causes symptoms of acute psychosis, deficits in spatial memory, and impairment of synaptic plasticity in rats. Hippocampus 18, 125–134. 10.1002/hipo.20367 17924525

[B64] Manahan-Vaughan D. Wildförster V. Thomsen C. (2008b). Rescue of hippocampal LTP and learning deficits in a rat model of psychosis by inhibition of glycine transporter-1 (GlyT1). Eur. J. Neurosci. 28, 1342–1350. 10.1111/j.1460-9568.2008.06433.x 18973561

[B65] Moghaddam B. (2003). Bringing order to the glutamate chaos in schizophrenia. Neuron 40, 881–884. 10.1016/s0896-6273(03)00757-8 14659087

[B66] Morris R. G. (2013). NMDA receptors and memory encoding. Neuropharmacology 74, 32–40. 10.1016/j.neuropharm.2013.04.014 23628345

[B67] Naie K. Manahan-Vaughan D. (2004). Regulation by metabotropic glutamate receptor 5 of LTP in the dentate gyrus of freely moving rats: relevance for learning and memory formation. Cereb. Cortex 14, 189–198. 10.1093/cercor/bhg118 14704216

[B68] Onslow A. C. E. Bogacz R. Jones M. W. (2011). Quantifying phase-amplitude coupling in neuronal network oscillations. Prog. Biophys. Mol. Biol. 105, 49–57. 10.1016/j.pbiomolbio.2010.09.007 20869387

[B69] Ota V. K. Noto C. Gadelha A. Santoro M. L. Ortiz B. B. Andrade E. H.. (2014). Evaluation of neurotransmitter receptor gene expression identifies GABA receptor changes: a follow-up study in antipsychotic-naïve patients with first-episode psychosis. J. Psychiatr. Res. 56, 130–136. 10.1016/j.jpsychires.2014.05.012 24935901

[B70] Patel J. Fujisawa S. Berényi A. Royer S. Buzsáki G. (2012). Traveling theta waves along the entire septotemporal axis of the hippocampus. Neuron 75, 410–417. 10.1016/j.neuron.2012.07.015 22884325 PMC3427387

[B71] Rajkai C. Lakatos P. Chen C.-M. Pincze Z. Karmos G. Schroeder C. E. (2008). Transient cortical excitation at the onset of visual fixation. Cereb. Cortex 18, 200–209. 10.1093/cercor/bhm046 17494059

[B72] Ringo J. L. Sobotka S. Diltz M. D. Bunce C. M. (1994). Eye movements modulate activity in hippocampal, parahippocampal, and inferotemporal neurons. J. Neurophysiol. 71, 1285–1288. 8201422 10.1152/jn.1994.71.3.1285

[B73] Rujescu D. Bender A. Keck M. Hartmann A. M. Ohl F. Raeder H.. (2006). A pharmacological model for psychosis based on N-methyl-D-aspartate receptor hypofunction: molecular, cellular, functional and behavioral abnormalities. Biol. Psychiatry 59, 721–729. 10.1016/j.biopsych.2005.08.029 16427029

[B74] Samudra N. Ivleva E. I. Hubbard N. A. Rypma B. Sweeney J. A. Clementz B. A.. (2015). Alterations in hippocampal connectivity across the psychosis dimension. Psychiatry Res. 233, 148–157. 10.1016/j.pscychresns.2015.06.004 26123450 PMC4784701

[B75] Seeman P. (1987). Dopamine receptors and the dopamine hypothesis of schizophrenia. Synapse 1, 133–152. 10.1002/syn.890010203 2905529

[B76] Seeman P. Bzowej N. H. Guan H. C. Bergeron C. Reynolds G. P. Bird E. D. (1987). Human brain D1 and D2 dopamine receptors in schizophrenia, Alzheimer’s, Parkinson’s and Huntington’s diseases. Neuropsychopharmacology 1, 5–15. 10.1016/0893-133x(87)90004-2 2908095

[B77] Sehatpour P. Dias E. C. Butler P. D. Revheim N. Guilfoyle D. N. Foxe J. J.. (2010). Impaired visual object processing across an occipital-frontal-hippocampal brain network in schizophrenia: an integrated neuroimaging study. Arch. Gen. Psychiatry 67, 772–782. 10.1001/archgenpsychiatry.2010.85 20679585 PMC4283949

[B78] Shirvalkar P. R. Rapp P. R. Shapiro M. L. (2010). Bidirectional changes to hippocampal theta-gamma comodulation predict memory for recent spatial episodes. Proc. Natl. Acad. Sci. U S A 107, 7054–7059. 10.1073/pnas.0911184107 20351262 PMC2872428

[B79] Snyder M. A. Gao W.-J. (2013). NMDA hypofunction as a convergence point for progression and symptoms of schizophrenia. Front. Cell. Neurosci. 7:31. 10.3389/fncel.2013.00031 23543703 PMC3608949

[B80] Steiner J. Walter M. Glanz W. Sarnyai Z. Bernstein H. G. Vielhaber S.. (2013). Increased prevalence of diverse N-methyl-D-aspartate glutamate receptor antibodies in patients with an initial diagnosis of schizophrenia: specific relevance of IgG NR1a antibodies for distinction from N-methyl-D-aspartate glutamate receptor encephalitis. JAMA Psychiatry 70, 271–278. 10.1001/2013.jamapsychiatry.86 23344076

[B81] Tort A. B. L. Komorowski R. W. Manns J. R. Kopell N. J. Eichenbaum H. (2009). Theta-gamma coupling increases during the learning of item-context associations. Proc. Natl. Acad. Sci. U S A 106, 20942–20947. 10.1073/pnas.0911331106 19934062 PMC2791641

[B82] Tsanov M. Manahan-Vaughan D. (2009). Visual cortex plasticity evokes excitatory alterations in the hippocampus. Front. Integr. Neurosci. 3:32. 10.3389/neuro.07.032.2009 19956399 PMC2786298

[B108] Vanderwolf C. H. (1969). Hippocampal electrical activity and voluntary movement in the rat. Electroencephalogr. Clin. Neurophysiol. 26, 407–418. 10.1016/0013-4694(69)90092-3 4183562

[B83] Velakoulis D. Wood S. J. Wong M. T. H. McGorry P. D. Yung A. Phillips L. (2006). Hippocampal and amygdala volumes according to psychosis stage and diagnosis: a magnetic resonance imaging study of chronic schizophrenia, first-episode psychosis and ultra-high-risk individuals. Arch. Gen. Psychiatry 63, 139–149. 10.1001/archpsyc.63.2.139 16461856

[B84] Vida I. Bartos M. Jonas P. (2006). Shunting inhibition improves robustness of gamma oscillations in hippocampal interneuron networks by homogenizing firing rates. Neuron 49, 107–117. 10.1016/j.neuron.2005.11.036 16387643

[B85] Vrajová M. Stastný F. Horácek J. Lochman J. Serý O. Peková S.. (2010). Expression of the hippocampal NMDA receptor GluN1 subunit and its splicing isoforms in schizophrenia: postmortem study. Neurochem. Res. 35, 994–1002. 10.1007/s11064-010-0145-z 20204507

[B86] Wiescholleck V. Manahan-Vaughan D. (2012). PDE4 inhibition enhances hippocampal synaptic plasticity *in vivo* and rescues MK801-induced impairment of long-term potentiation and object recognition memory in an animal model of psychosis. Transl. Psychiatry 2:e89. 10.1038/tp.2012.17 22832854 PMC3309535

[B87] Wiescholleck V. Manahan-Vaughan D. (2013a). Long-lasting changes in hippocampal synaptic plasticity and cognition in an animal model of NMDA receptor dysfunction in psychosis. Neuropharmacology 74, 48–58. 10.1016/j.neuropharm.2013.01.001 23376021

[B88] Wiescholleck V. Manahan-Vaughan D. (2013b). Persistent deficits in hippocampal synaptic plasticity accompany losses of hippocampus-dependent memory in a rodent model of psychosis. Front. Integr. Neurosci. 7:12. 10.3389/fnint.2013.00012 23508474 PMC3597980

[B89] Wöhrl R. Haebler D. Heinemann U. (2007). Low-frequency stimulation of the direct cortical input to area CA1 induces homosynaptic LTD and heterosynaptic LTP in the rat hippocampal-entorhinal cortex slice preparation. Eur. J. Neurosci. 25, 251–258. 10.1111/j.1460-9568.2006.05274.x 17241286

[B90] Yin D. Chen Y. Sathyamurthy A. Xiong W. Mei L. (2012). Synaptic dysfunction in schizophrenia. Adv. Exp. Med. Biol. 970, 493–516. 10.1007/978-3-7091-0932-8_22 22351070

[B91] Zhang Y. Yoshida T. Katz D. B. Lisman J. E. (2012). NMDAR antagonist action in thalamus imposes delta oscillations on the hippocampus. J. Neurophysiol. 107, 3181–3189. 10.1152/jn.00072.2012 22423006 PMC3378362

[B92] Zylla M. M. Zhang X. Reichinnek S. Draguhn A. Both M. (2013). Cholinergic plasticity of oscillating neuronal assemblies in mouse hippocampal slices. PLoS One 8:e80718. 10.1371/journal.pone.0080718 24260462 PMC3832478

